# Tear Film Conditions and Depression Symptoms in Persons with Obesity

**DOI:** 10.3390/diagnostics14161768

**Published:** 2024-08-14

**Authors:** Anabel Sanchez-Sanchez, MaGuadalupe Leon-Verdin, Sabino Chavez-Cerda, Claudia Martinez-Cordero

**Affiliations:** 1 Instituto Nacional de Astrofísica Óptica y Electrónica, Puebla 72840, Mexico; optiani@hotmail.com (A.S.-S.); sabino@inaoep.mx (S.C.-C.); 2 Campus León, Universidad de Guanajuato, León 37320, Mexico; lupitalv2001@gmail.com; 3 Servicios de Salud del Instituto Mexicano del Seguro Social para el Bienestar (IMSS-BIENESTAR) Hospital Regional de Alta Especialidad del Bajío, León 37544, Mexico

**Keywords:** obesity, depression, tear film, BUT, Schirmer

## Abstract

Most persons with obesity who have undergone gastric bypass surgery present depressive symptoms. Depression and anxiety have been associated with tear film disorders. This study aimed to investigate whether there is a correlation between tear film conditions and depression symptoms in patients subjected to bariatric surgery. The participants completed a Patient Health Questionnaire to detect depression symptoms. The break-up time and Schirmer test were subsequently applied; the measurements were performed three times, and the average time was subsequently recorded. The results revealed that the Schirmer test score and PH-9 score were negatively correlated, but this was not the case for the break-up time test. Depression symptoms may correlate with lower tear production from the principal tear gland, but they may not have the same effect on meibomian gland production in adults undergoing bariatric surgery. In addition to routine control, bariatric surgery patients should be periodically evaluated by an ophthalmologist and/or psychologist who is aware of potential comorbidities. Furthermore, the observed association between depression symptoms and tear deficiencies highlights the importance of further investigations to gain a better understanding of these mechanisms.

## 1. Introduction

Most patients who have undergone gastric bypass surgery present depressive symptoms that gradually increase beyond 24 months after surgery [1]. Depression and anxiety have been associated with tear film disorders [2,3,4,5,6]. The most common tool for screening for depression symptoms is the Patient Health Questionnaire 9 (PHQ-9) [7]. The Schirmer test and break-up time (BUT) test evaluate different properties of the tear film: the Schirmer test evaluates the quantity of the tear aqueous component, which is produced mainly by the lacrimal gland, whereas the BUT evaluates the tear lipid component, which defines the tear film stabilization time produced mainly by the meibomian glands [8].

**Figure 1:**
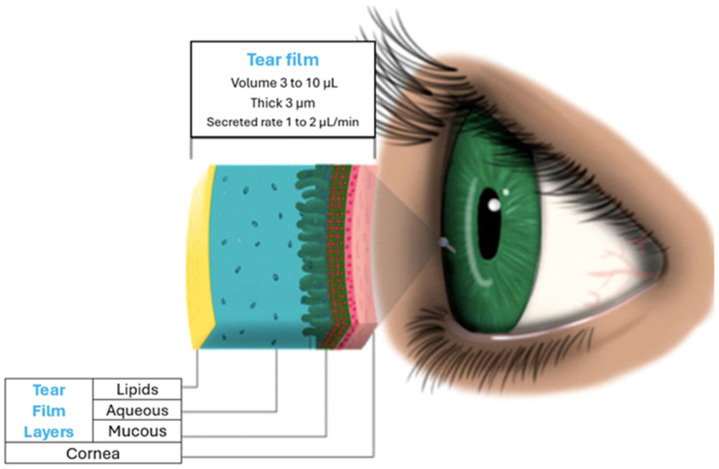
The traditional representation of tear layers and tear characteristics.

The study of the lacrimal system includes anatomical structures involved in three processes: the production, distribution, and excretion of the tear film. During the tear film production process, three layers are involved: lipids, aqueous liquids, and mucous (Figure 1). According to its most recent definition by Gayton, these layers are interactive gels of hydrated mucin with protein-associated lipids distributed throughout the gel. The tear film plays an important role in the health and development of the functions of the anterior segment of the eyeball. Among these, it contributes optically by forming a regular film on the cornea while also lubricating it; it defends the eye from infections and preserves the health of the cornea and conjunctiva owing to its enzyme content and pH (7.1–7.8). Moreover, it is the first barrier to overcome for the entry of drugs into the eyeball through this route, since when drugs are applied, they are diluted in their components, reducing the concentrations that can reach the corneal basal epithelium. Modifications in its production give rise to ailments that can range from deficiencies in some of its layers, which are treatable with eye drops, to advanced degrees that result in corneal ulcers or blindness that require more invasive and costly treatment [9,10].

The tear film layers are secreted by the following glands: meibomian, sebaceous Zeis, and Sudoriparo Moll, which are located behind the follicles of the eyelashes at the edge of the eyelids and are responsible for forming the lipid layer, the main function of which is to stabilize the tear film and prevent its evaporation [11]. The main lacrimal gland, Wolfring, and Krause glands secrete components of the aqueous layer, and their role is to clean the cornea, allowing the mobility of the palpebral conjunctiva on the cornea and contributing to optical quality. Goblet cells, the glands of Manz, and the crypts of Henle of the conjunctiva are responsible for producing the hydrophilic mucous layer that contributes to the adhesion of the tear film to the villi of the corneal epithelium [12].

The production of these glands is regulated by the autonomic nervous system through two linked reflex arcs: (1) hydrodynamic: responsible for flickering and sensitivity; (2) secretion: regulates glandular production and the different layers.

The main lacrimal gland receives innervation from cranial nerves V and VII and is classified as a combined or mixed type. This classification defines cranial nerves as having very little relation to motor function. Nerve V is the longest cranial nerve and the most important sensory nerve in the face. It has three branches: the ophthalmic, maxillary, and mandibular (V3) branches. The ophthalmic branch (which defines corneal sensitivity) is sensitive, originates in the upper part of the face, surface of the eyeballs, lacrimal gland, upper nasal mucosa, frontal sinuses and ethmoid. In addition, it flows into the bulge. The facial nerve (VII) has a sensory function in taste and is the most important motor nerve of the muscles of the face. It has five branches: temporal, zygomatic, buccal, mandibular and cervical. Within its motor functions are facial expressions, tear secretion (the parasympathetic efferent arm controls the secretory glands), saliva, and nasal and buccal mucus production [11].

Thus, the purpose of this work was to investigate whether there is a correlation between tear film conditions evaluated by the Schirmer test and BUT and depression symptoms identified through the PHQ-9 in patients subjected to bariatric surgery as a clinical treatment in a public hospital to overcome severe obesity. This study is the first in Mexico to explore the relationship between symptoms of depression and tear film characteristics in a group of people who underwent bariatric surgery.

## 2. Materials and Methods

### Sample

An ethical committee (CI/HRAEB/044/2020) approved this pilot study, and the patients signed a written consent form for participation. A sample of 29 people was calculated via a 95% confidence interval and a significance threshold of 10% [13]. We invited all patients from the surgery service of a public hospital who underwent Roux-en-Y gastric bypass (RYGB) to participate. The selection criteria were male and female patients aged 20–65 years with a postsurgical duration of at least two years. The exclusion criterion was patients having associated systemic disease, specifically diabetes or metabolic syndrome, due to the drug’s effects on the lacrimal glands and/or corneal diseases or on the anterior segment of the eye. None of the participants were under pharmacological depression treatment because none were known to live with severe depression symptoms. Overall, 30 patients were eligible and indicated their willingness to underdo this protocol. The participants had a mean age of 48.5 years and a mean BMI of 32.7 kg/m^2^.

Depressive symptoms were evaluated with the Patient Health Questionnaire (PHQ-9), which evaluates the presence and severity of depressive symptoms according to the criteria of the Handbook of Diagnostic and Statistical of Mental Disorders; the PHQ-9 generates a scale from 2 to 27, and a score of up to 5 suggests the presence of depressive symptoms [14,15]. The Schirmer test and BUT were subsequently applied by specialists.

**Figure 2:**
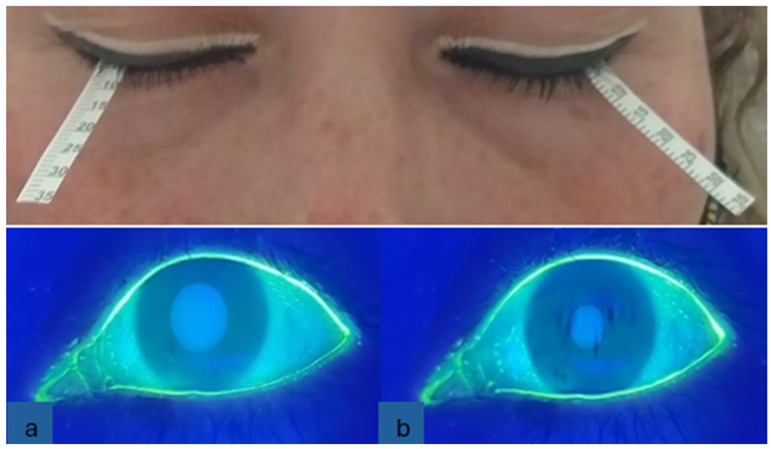
Tear film tests. Top, Schirmer. Bottom, BUT: (**a**) dyeing with fluorescein; (**b**) black spots.

We evaluated tear films with the Schirmer test and BUT. For the Schirmer test, without anesthesia, special strips (5 × 35 mm) were gently placed over the lower eyelid outer third margin of each eye simultaneously without touching the cornea. The eyes of the patients were kept closed, and the chronometer was started. During this time, ocular movements were minimally controlled. The strips were removed when the chronometer was used for five minutes, and the strip wetting was measured in millimeters. For the BUT, one drop of sodium fluorescein (1 mg) was applied to the upper conjunctiva, after which the patients were allowed to blink. Once the substance was homogeneously distributed on the anterior surface via a Burton lamp, the patient was asked to close and open the eyes three times, and the chronometer was activated at the same time. Chronometry was stopped when black spots or screams appeared in the tear film with fluorescein. The measurements were made three times, and the average time was subsequently recorded. Figure 2 shows the placement of the Schirmer strips on the outer third margin of each eye. In the same figure at the bottom, the BUT (a) shows the beginning with the regular distribution of fluorescein and (b) the end of the test with the appearance of black spots.

## 3. Results

**Figure 3:**
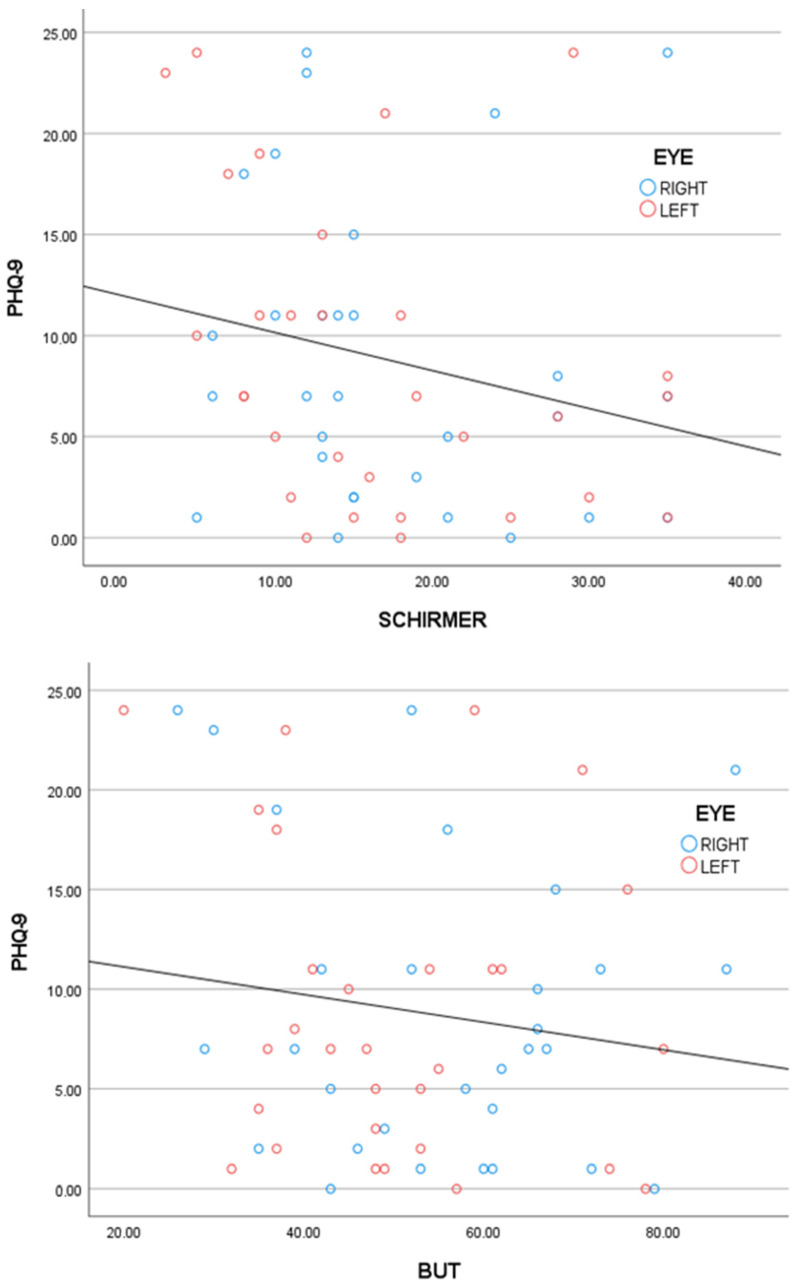
Top, PHQ-9 score vs. Schirmer score. Bottom, PHQ-9 score vs. BUT score. Both tear film parameters had negative slopes and negative correlations with the PHQ-9 score. The results revealed that the Schirmer test and the PHQ-9 score had a statistically significant *p* value for a negative correlation but not for the BUT.

A total of 30 volunteers fulfilled the selection criteria. Table 1 shows the characteristics of the patients. For the PHQ-9 (Cronbach’s alpha = 0.912), the mean score was 8.8 ± 7.5. The average tear film test score and correlation with the PHQ-9 score obtained from Spearman’s rho statistics are shown in Table 2. The results revealed that the Schirmer test score and the PQH-9 score were negatively correlated, but this was not the case for the BUT. The measurements were made three times, and the average time was recorded. Additionally, a GLM (general linear model) was used to test the effect of the PQH-9 score adjusted by the eye side (left or right) for the Schirmer score (β = −0.27, *p* value = 0.08) and the BUT score (β = −0.03, *p* value = 0.26); see Figure 3. In the models, there were no significant interactions or eye side effects (*p* value > 0.1). All the data analyses and graphics were performed and created, respectively, via SPSS 27 software.

## 4. Discussion

Tear secretion is regulated through the autonomous nervous system. Therefore, tear film disorders may be caused by the dysfunction of tear-secreting glands, an anomaly in their neuronal circuit, or both [16]. When autonomic fibers are terminated, they reach the submandibular and sublingual salivary glands, lacrimal glands, and nasal and palatine glands. In addition, tear secretion controls flickering activity, which is important for the distribution and drainage of the tear film [11].

Secretion by the main lacrimal gland is regulated by a neuronal response in two phases: 1. the afferent nerves that come from the cornea and conjunctiva; 2. the efferent sympathetic and parasympathetic nerves that innervate the gland, its secretory cells and its excretory ducts. Owing to the importance of the tear volume, two of the most studied types of glands contribute to the tear film: the main lacrimal gland and the meibomian glands. In the meibomian glands in humans, a dense mesh of unmyelinated nerve fibers (nerve plexus) has been described around the acini. The innervation of the meibomian glands is carried out through this mesh with several origins: the main parasympathetic nerves from the pterygopalatine ganglion, the sympathetic nerves from the superior cervical ganglion and the sensory fibers from the trigeminal ganglion [12].

First, low BUT values possibly indicate that the meibomian glands are sensitive to depressive states related to lower parasympathetic ANS activity and predominant innervation of these glands [12]. Additionally, the functions of the lacrimal and meibomian glands could be affected by autonomic dysfunction, which is reported after bariatric surgery [17], during which an increase in subsequent depressive symptoms occurs [1]. These results suggest that depression leads to decreased tear production from the principal tear gland but that there seem to be different modifications to meibomian gland production. However, the Schirmer scores are normal [12], whereas the BUT values are below the normal parameters [17]. Second, afferent stimulation activates the efferent response, resulting in the secretion of electrolytes, water, and proteins through the excretory ducts to the ocular surface, that surrounds the gland in liquid. Sensory input is processed in the brain in the lacrimal nucleus and includes emotional input, among other inputs. The negative correlation between the Schirmer score and the PHQ-9 score could be related to a modification in the motor innervation of the lacrimal gland from the facial nerve during depression [18]. Third, the fibers of human meibomian glands are sensitive to acetylcholinesterase, which is a component of the cholinergic parasympathetic nervous system. The main function of acetylcholinesterase is to rapidly inactivate acetylcholine. Acetylcholine is the main neurotransmitter of the autonomic nervous system that favors body secretions. When acetylcholinesterase slows acetylcholine, gland production decreases [18]. Thus, low BUTs possibly indicate that the meibomian glands are sensitive to depressive states related to parasympathetic ANS activity and the predominant innervation of these glands [12] or that these low BUTs could be modified by nutritional factors, as has been reported in other studies [19,20,21].

## 5. Conclusions

Our study demonstrated that the presence of depression symptoms is related to aqueous layer production during tear film production in adults undergoing bariatric surgery. In addition to routine control, bariatric surgery patients should be periodically evaluated by an ophthalmologist and psychologist who is aware of potential comorbidities. Furthermore, the observed association between depression symptoms and tear deficiencies highlights the importance of further investigations to gain a better understanding of these mechanisms.

## Figures and Tables

**Table 1:** Characteristics of patients.

Variables	Age	Weight	Height	BMI	Qx time	Glucose
Units	Years	kg	m	kg/m^2^	years	mg/dL
Mean/SD	48.5 ± 9	81 ± 16	1.6 ± 0.1	32.7 ± 5.5	7.4 ± 2	89.4 ± 7.8
Range	33–65	51.5–114.7	1.4–1.7	23.8–48.7	2.5–12.0	74–109

**Table 2:** The average values of the tear film tests and correlation with the PHQ-9 score.

Tests	Units	Mean/SD	PHQ-9 (Correlation)
Schirmer	Millimeters (mm)	17.0 ± 9.1	−0.31 *
BUT	Seconds (s)	5.2 ± 1.6	−0.11

* Statistically significant.

## Data Availability

Data analyzed during this study are included in this article. Further inquiries can be directed to the corresponding author.

## References

[1] White M.A., Kalarchian M.A., Levine M.D., Masheb R.M., Marcus M.D., Grilo C.M. (2015). Prognostic Significance of Depressive Symptoms on Weight Loss and Psychosocial Outcomes Following Gastric Bypass Surgery: A Prospective 24-Month Follow-Up Study. Obes. Surg..

[2] Weatherby T.J.M., Raman V.R.V., Agius M. (2019). Depression and dry eye disease: A need for an interdisciplinary approach?. Psychiatr. Danub..

[3] Kitazawa M., Sakamoto C., Yoshimura M., Kawashima M., Inoue S., Mimura M., Tsubota K., Negishi K., Kishimoto T. (2018). The Relationship of Dry Eye Disease with Depression and Anxiety: A Naturalistic Observational Study. Transl. Vis. Sci. Technol..

[4] Chang K.-J., Wu H.-Y., Chiang P.-H., Hsu Y.-T., Weng P.-Y., Yu T.-H., Chen Y.-H., Dai H.-J., Tsai H.-Y., Chang Y.-J. (2024). Decoding and reconstructing disease relations between dry eye and depression: A multimodal investigation comprising meta-analysis, genetic pathways and Mendelian randomization. J. Adv. Res..

[5] Liu Z., Sun S., Sun X., Wu Y., Huang Y. (2022). Differences of Anxiety and Depression in Dry Eye Disease Patients According to Age Groups. Front. Psychiatry.

[6] Tsai C.Y., Jiesisibieke Z.L., Tung T.H. (2022). Association between dry eye disease and depression: An umbrella review. Front. Public Health.

[7] Levis B., Benedetti A., Thombs B.D. (2019). Accuracy of Patient Health Questionnaire-9 (PHQ-9) for screening to detect major depression: Individual participant data meta-analysis. BMJ.

[8] Shapiro A., Merin S. (1979). Schirmer test and break-up time of tear film in normal subjects. Am. J. Ophthalmol..

[9] Dartt D.A., Willcox M.D. (2013). Complexity of the tear film: Importance in homeostasis and dysfunction during disease. Exp. Eye Res..

[10] Van Der Vaart R., Weaver M.A., Lefebvre C., Davis R.M. (2015). The association between dry eye disease and depression and anxiety in a large population-based study. Am. J. Ophthalmol..

[11] Dartt D.A. (2009). Neural regulation of lacrimal gland secretory processes: Relevance in dry eye diseases. Prog. Retinal Eye Res..

[12] Knop E., Knop N., Millar T., Obata H., Sullivan D.A. (2011). The international workshop on meibomian gland dysfunction: Report of the subcommittee on anatomy, physiology, and pathophysiology of the meibomian gland. Invest. Ophthalmol. Vis. Sci..

[13] Viechtbauer W., Smits L., Kotz D., Budé L., Spigt M., Serroyen J., Crutzen R. (2015). A simple formula for the calculation of sample size in pilot studies. J. Clin. Epidemiol..

[14] Spitzer R.L., Kroenke K., Williams J.B. (1999). Validation and utility of a self-report version of PRIME-MD: The PHQ primary care study. Primary Care Evaluation of Mental Disorders. Patient Health Questionnaire. JAMA.

[15] Kroenke K., Spitzer R.L., Williams J.B. (2001). The PHQ-9: Validity of a brief depression severity measure. J. Gen. Intern Med..

[16] Meng I.D., Kurose M. (2013). The role of corneal afferent neurons in regulating tears under normal and dry eye conditions. Exp. Eye Res..

[17] Rodriguez R.J., Badin A., Panday M., Anderson A., Pillarisetti J. (2021). Autonomic dysfunction following bariatric surgery. J. Am. Coll. Cardiol..

[18] Nellis J.C., Ishii M., Byrne P.J., Boahene K.D.O., Dey J.K., Ishii L.E. (2017). Association Among Facial Paralysis, Depression, and Quality of Life in Facial Plastic Surgery Patients. JAMA Facial Plast Surg..

[19] Jalbert I. (2013). Diet, nutraceuticals and the tear film. Exp. Eye Res..

[20] Kawashima M., Kawakita T., Inaba T., Okada N., Ito M., Shimmura S., Watanabe M., Shinmura K., Tsubota K. (2012). Dietary lactoferrin alleviates age-related lacrimal gland dysfunction in mice. PLoS ONE.

[21] Sánchez-Sánchez A.S., Rodríguez-Murguía N., Martinez-Cordero C., Chávez-Cerda S. (2020). Protein Diet in Bariatric Patients Could Modify Tear Film. Obes. Surg..

